# Born in Brussels screening tool: the development of a screening tool measuring antenatal psychosocial vulnerability

**DOI:** 10.1186/s12889-021-11463-8

**Published:** 2021-08-06

**Authors:** Kelly Amuli, Kim Decabooter, Florence Talrich, Anne Renders, Katrien Beeckman

**Affiliations:** 1 Faculty of Medicine and Pharmacy Department of Public Health, Nursing and Midwifery Research Group, Vrije Universiteit Brussel - Campus Jette, Brussel, BE Belgium; 2 Department of Nursing and Midwifery research group (NUMID), Universitair Ziekenhuis Brussel, Laarbeeklaan 101 1090 Brussel, Jette, BE Belgium; 3 Verpleeg- en vroedkunde, Centre for Research and Innovation in Care, Midwifery Research Education and Policymaking (MIDREP), Universiteit Antwerpen, Antwerp, Belgium

**Keywords:** Antenatal, Pregnancy, Psychosocial, Vulnerability, Screening, Questionnaire

## Abstract

**Background:**

Antenatal psychosocial vulnerability is a main concern in today’s perinatal health care setting. Undetected psychosocially vulnerable pregnant women and their unborn child are at risk for unfavourable health outcomes such as poor birth outcomes or mental state. In order to detect potential risks and prevent worse outcomes, timely and accurate detection of antenatal psychosocial vulnerability is necessary. Therefore, this paper aims to develop a screening tool ‘the Born in Brussels Screening Tool (ST)’ aimed at detecting antenatal psychosocial vulnerability.

**Methods:**

The Born in Brussels ST was developed based on a literature search of existing screening tools measuring antenatal psychosocial vulnerability. Indicators and items (i.e. questions) were evaluated and selected. The assigned points for the answer options were determined based on a survey sent out to caregivers experienced in antenatal (psychosocial) vulnerability. Further refinement of the tool’s content and the assigned points was based on expert panels’ advice.

**Results:**

The Born in Brussels ST consists of 22 items that focus on 13 indicators: communication, place of birth, residence status, education, occupational status, partner’s occupation, financial situation, housing situation, social support, depression, anxiety, substance use and domestic violence. Based on the 168 caregivers who participated in the survey, assigned points account between 0,5 and 4. Threshold scores of each indicator were associated with adapted care paths.

**Conclusion:**

Generalied and accurate detection of antenatal psychosocial vulnerability is needed. The brief and practical oriented Born in Brussels ST is a first step that can lead to an adequate and adapted care pathway for vulnerable pregnant women.

**Supplementary Information:**

The online version contains supplementary material available at 10.1186/s12889-021-11463-8.

## Introduction

Pregnant women are considered psychosocially vulnerable when facing one or more unfavourable personal and environmental situation(s) [1, 2] (for e.g. health problems, psychological distress, substance abuse, low economic status, poor housing situation, domestic violence, poor social support or others). As a result, a psychosocially unfavourable situation can affect a pregnant woman’s access to care [3] or lead to adverse perinatal outcomes [4] such as low birth weight [5], preterm birth [5], maternal mortality [6], morbidity [7], excessive gestational weight gain [8], depression or anxiety [9].

Currently, antenatal psychosocial vulnerability stays undetected due to a lack of systematic screening [10]. Elements such as domestic violence, social isolation, poverty or depression that might be present during pregnancy are often not visible and as such not (correctly) discussed or interpreted during a consultation [2, 11]. De Waal et.al analysed that only 5.3% of pregnant women are detected as being vulnerable during an antenatal consultation, compared to 27% when using a screening tool. Hence, unsystematic screening for symptoms of depression can result in missing out 3 out of 4 pregnant women otherwise detected with the Edinburgh Postnatal Depression Scale (EPDS) [12]. In addition, a retrospective study showed that screening of psychosocial indicators could result in less adverse pregnancy outcomes [13]. The use of a standardised screening tool, in which these and other sensitive elements are incorporated, can therefore open a conversation on sensitive topics and lead to an increased detection of antenatal psychosocial vulnerability [14–16].

Although highly recommended by health institutions [17, 18], to date, few screening tools exist that measure the multidimensionality of antenatal psychosocial vulnerability [16, 19, 20]. The existing screening tools differ in content, depending on which indicators of vulnerability are included, and vary in length. Extensive questionnaires can offer a broad view of vulnerability; however, it can be a burden for the patient [21] or the health care provider to complete them [2, 22]. Moreover, it can be challenging for the caregiver to screen in a non-stigmatising way when discussing sensitive topics such as mental health [22].

There is a need for an inclusive screening tool in Belgium and specifically the Brussels Metropolitan Region. This since, in Brussels, 18% of reproductive aged women have depressive feelings [23], about 17% are single mothers, 45% of mothers are not active on the labour market [24] and 41.5% new-borns are born in a household living under the poverty line [25]. Given the importance of these problems, the Belgian federal government started the project Born in Brussels. This four-year project of the Belgium National Institute for Health and Disability Insurance (NIHDI) has as main objective, the creation of an uniform care path for psychosocial vulnerable pregnant women in Brussels. For this purpose, there is a need for a concise screening tool that acknowledges the multidimensionality of antenatal psychosocial vulnerability. This paper reports on the development of an antenatal psychosocial vulnerability-screening tool in a metropolitan area, the Born in Brussels Screening Tool (ST), based on existing screening tools and experts’ opinion.

## Methods

The Ethics Committee of UZ Brussels has approved this study (B.U.N. 143201941861) on December 18 2019.

**Figure 1:**
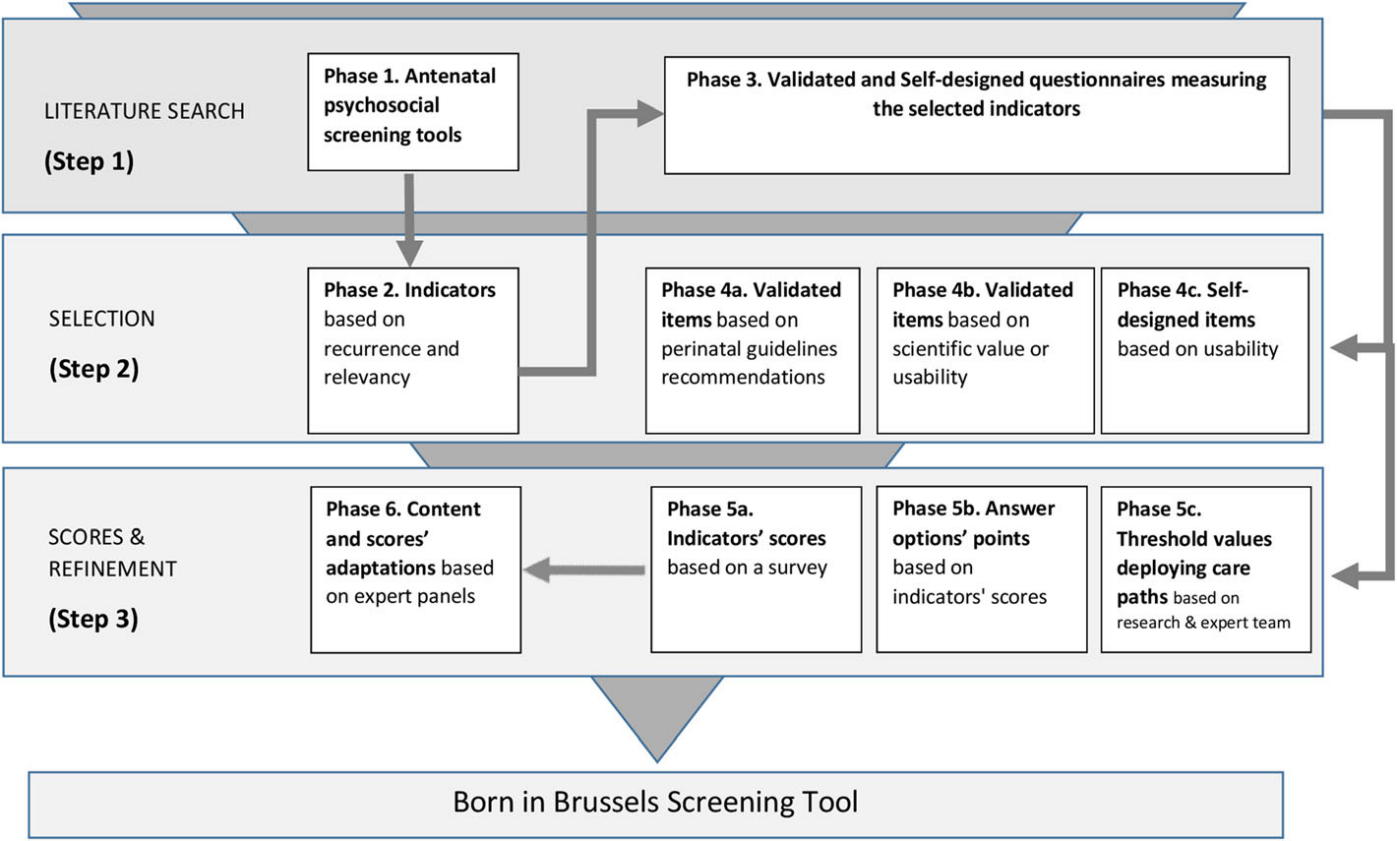
**Fig. 1** Illustrates the different steps and phases taken for the development of the Screening Tool. During phases 1 and 3, a literature search was done (step 1). The results from the literature search were analysed and selected based on selection criteria in phases 2 and 4 (step 2). This selection step resulted in indicators and items that form the Born in Brussels Screening Tool. In phase 5 and 6 scores, points, treshold values and refinements were determined (step 3)

Several steps were taken to develop the Born in Brussels ST (Fig. 1). A first step was a literature search to identify existing screening tools (or questionnaires) measuring antenatal psychosocial vulnerability. These tools were further analysed to select the most occurring indicators and the most relevant items (i.e. questions) that will constitute the Born in Brussels ST (step 2). Next, thresholds were determined. The score of an indicator that is above its threshold value would deploy the corresponding care path. Subsequently to the construction of the Born in Brussels ST, the research team refined the content and points of the tool based on an expert panel’s advice (step 3).

### Literature search – step 1

We performed a literature search of existing antenatal psychosocial screening tools measuring at least two psychosocial indicators during the antenatal period. We then listed the common screened indicators, their frequency and the types of questionnaires (i.e. validated or self-designed questionnaires) that are included in their screening tools. Medical indicators were also included in order to determine their possible necessity in psychosocial screening tools (Fig. 1: phase 1).

After we selected the most recurrent and relevant indicators (see step 2), we further searched for validated or self-designed questionnaires other than the ones found in the screening tools. This, for each indicator (Fig. 1: phase 3).

Articles and grey literature (i.e. governmental and institutional reports, theses and dissertations, guidelines, unpublished conference articles or documents) were consulted on the PubMed database, Google, Google Scholar or from documentation of institutions. Search terms such as ‘pregnancy’, ‘antenatal’, ‘vulnerabilities’, ‘social inequality’, ‘psychosocial’, ‘psychosocial deprivation’, ‘questionnaire, screening’, indicators-specific term (e.g. ‘depression’) and a time filter (i.e. from 2010 to 2018) were used to include screening tools  cited or used in papers published during the last 10 years. This, in order to gather the most recent screening tools.

### Selection of the indicators and items for the Born in Brussels ST – step 2

All screening tools found in the literature search were evaluated to select indicators that will form the Born in Brussels ST. We first made a selection of recurring indicators and then established a selection based on relevant indicators. Recurring indicators are those that appear at least 25% of the total tools found. Relevant indicators are those considered by the research team and experts as applicable for the project goal (i.e. the psychosocial current situation of a metropolitan pregnant population) regardless of whether the indicator shows a low (i.e. less than 25%) or high (i.e. more than 25%) recurrence (Fig. 1: phase 2).

Next, we evaluated the validated and self-designed items found in phase 3. We based our item’s selection on found perinatal guidelines’ recommendations, scientific value (i.e. the sensitivity, specificity, predictive value, validity and reliability were analysed), usability (i.e. number of items, understandable context) and relevancy (i.e. for a pregnant population) (Fig. 1: phase 4).

### Born in Brussels ST: assigned points, associated care paths’ thresholds and ST refinement – step 3

In step 3, the points and the threshold scores for the deployment of the associated care paths were determined. We first determined the weight of each indicator to assign them a score. Points were then assigned to the tool’s answer options and care paths' threshold scores were determined.

Scores (here defined as the total number of points) per indicator were determined using a survey sent to caregivers with expertise in prenatal vulnerability (i.e. midwives, social nurses, social workers, general practitioners, psychiatrists, psychologists, and other medical or non-medical professionals), also considered as the target users of the ST. The caregivers’ point of view was aimed at determining the level of psychosocial vulnerability of the indicators. The survey asked participants to weigh the retained indicators between 1 and 10 (1 meaning a less determining factor of vulnerability and 10 meaning a very determining factor of vulnerability). Based on the weights participants assigned to the indicators, we calculated the mean for each indicator. Based on the quartile distribution, these means were divided into four categories, enabling us to assign a score to each indicator. A score was attributed to each category: category one, which contains the lowest means, received a score of 1 whereas category four with the highest means received a score of 4. Summarised, a score of 1, 2, 3 or 4 could be attributed to an indicator (Fig. 1: phase 5a).

Based on the score assigned to an indicator, we assigned points to the answer options of each indicator-specific item. For an item where its indicator received a score of 4 for example, the points of the answer options ranged between 0 and 4. The distribution of the points (e.g. 1, 2, 3, 4 or 0, 2, 2, 4) was either replicated as the same points’ distribution of the item that has been included in the tool or determined by the research team who relied partly on the severity of the answer option for the distribution of the points (Fig. 1: phase 5b).

Finally, the research and expert team determined the indicators threshold scores necessary to deploy their associated care path (Fig. 1: phase 5c).

In addition, based on caregivers’ expertise, the content and points of the Born in Brussels ST were refined (Fig. 1: phase 6). Suggestions were made through the comment section of the above-mentioned survey or during the expert panels—composed of psychologists, paediatric psychiatrists, social workers, experts by experience in poverty, midwives and care coordinators from different organisations and hospitals that work in the Brussels Metropolitan Region—,organised as part of the Born in Brussels project.

## Results

### Literature search of screening tools – phase 1 of step 1

Twenty-two screening tools that focus on at least two elements of antenatal psychosocial vulnerability were found (Table 1). Of these, 14 resulted from the PubMed literature search and 8 from the grey literature study (i.e. 6 tools from Belgium and 2 from France). The number of items of the screening tools varied between 6 and 70 items. Some screening tools included validated questionnaires such as the Edinburgh Postnatal Depression Scale (EPDS), the Social Support Questionnaire - Short Form (SSQ-6) and others (Table 1).

**Table 1:** Literature review of antenatal psychosocial screening tools and the selection of indicators defining antenatal psychosocial vulnerability (phase 1 and 2 in Fig. 1)

LITERATURE REVIEW			SOCIO DEMOGRAPHIC/ ECONOMIC STATUS								MEDICAL				MENTAL STATE				SOCIAL SITUATION		SUBSTANCE USE			OTHER
Psychosocial instruments	Country (source ¥)	N° of Items counted	Communication	Age	Place of birth	Education	Marital status	Occupation	Financial situation	Housing situation	Medical/ Obstetrics	Medication use	Unwanted/ unplanned pregnancy	Late follow-up	Depression	Anxiety	Psychological history	Stress	Social support	Violence	Alcohol	Drugs	Tobacco	Other
**UZ Brussels (1)**	BE (G)	10	**X**		**X**	**X**	**X**	**X**	**X**												**X**	**X**	**X**	**X**
**C.D.V.P base** ***(& C.D.V.P approfondi)*** **(2)**	BE (G)	17 (28)		X		X	X	*(X)*	X		X	X	X	X	X	X	X	*(X)*	X	X	X	X	*(X)*	**X**
**Kind&Gezin** **Kansarmoede criteria**	BE (G)	6				X		X	X	X	X													**X**
**Fiche socio-médicale ONE**	BE (G)	14	X		X	X	X	X		X		X	X					X			X	X	X	**X**
**Psychosocial assessment &** ***(mental health screening protocol*) Van Damme et al. (3)**	BE (G)	28							X	X	X		X		*(Whooley & EPDS)*	*(GAD-2 & GAD- 7)*	X		OSLO-3	X	X	X	X	**X**
**EMBRACE (4)**	BE (G)	10	X	X		X			X	X			X	X	X	X	X	X	X	X	X	X	X	**X**
**EPICES(5)**	FR (P)	11					X		X	X									X					**X**
**AQ- Lille-Roubaix(6)**	FR (P)	17													EPDS				SSQ-6 + X	X	T-ACE	X	HSI	
**AQ-Languedoc Roussillon(7)**	FR (G)	16							X	X		X			EPDS				SSQ-6 + X	X	T-ACE	X	HSI	
**AQ-GEGA(8)**	FR (G)	18							X	X		X			EPDS				SSQ-6 + X	X	X	X	HSI	**X**
**Mind2Care(9)**	NL (P)	64		X	X	X	X	X	X	X		X	X	X	EPDS	WDEQ-A	X		X	X	X	X	X	**X**
**R4U(10)**	NL (P)	70	X	X	Ethnicity	X	X	X	X	X	X	X	X	X			X		X	X	X	X	X	X
**KINDEX(11)**	DE (P)	34		X	X		X		X	X	X						X	PSS-4		X	X	X	X	X
**PPP(12)**	USA (P)	44							X	X								X	X	X	X	X		X
**PRO(13)**	USA (P)	58								X					PHQ-9				MSSI	AAS	NSDUH	NSDUH	NSDUH	X
**ASAPS(14)**	USA (P)	28								X					CES-D	STAI		STR						X
**Antenatal psychosocial assessment(15)**	AUS (P)	unspecified													X		X	X	X	X	X	X		
**ANRQ(16)**	AUS (P)	12													X	X	X	X	X	X				
**ALPHA(17)**	CA (P)	35										X	X	X	X	X	X		X	WAST	CAGE+ X	X		X
**ARPA(18)**	AUS (P)	12															X	X	X	X				X
**PRQ(19)**	AUS (P)	18													X	X	X	X	X	X				X
**CAN-M(20)**	UK (P)	unpsecified (26 domains)	X			X			X	X					X	X		X	X	X	X	X		X
Selection based on recurrence (*)			**5**	**5**	**5**	8*	8*	6*	13*	15*	**5**	7*	7*	**5**	14*	10*	12*	11*	18*	18*	17*	17*	12*	**18**
**Selection based on relevancy (Yes)**			**Yes**	**No**	**Yes**	**Yes**	**Yes**	**Yes**	**Yes**	**Yes**	**No**	**No**	**No**	**No**	**Yes**	**Yes**	**No**	**No**	**Yes**	**Yes**	**Yes**	**Yes**	**Yes**	**No**
**Selected indicators for the Born in Brussels tool**			**Communication**		**Place of birth**	**Education**	**Marital status**	**Occupation**	**Financial situation**	**Housing situation**					**Depression**	**Anxiety**			**Social support**	**Violence**	**Alcohol**	**Drugs**	**Tobacco**	

¥ = literature from PubMed (=P) or Grey literature (=G); X = self-designed item(s)

* = appear more than 25%

(References can be found in additional file 1)

*C.D.V.P* Carnet de dépistage de la Vulnérabilité Périnatale; *ONE* Office de la Naissance et de l’Enfance; *EPICES* Évaluation de la Précarité et des Inégalités de santé dans les Centres d’Examens de Santé); *AQ* Auto-Questionnaire; *GEGA* Groupe d’étude Grossesse et addiction. *R4U* Rotterdam Reproductive Risk Reduction (R4U) scorecard; *PPP* Prenatal Psychosocial Profile; *PRO* Prenatal Risk Overview; *ASAPS* Abbreviated Scale for the Assessment of Psychosocial Status; *ANRQ* AnteNatal Risk Questionnaire; *ALPHA* AntenataL Psychosocial Health Assessment; *ARPA* Australian Routine Psychosocial Assessment; *PRQ* Pregnancy Risk Questionnaire; *CAN-M* Camberwell Assessment of Need – Mothers. *EPDS* Edinburgh Postnatal Depression Scale; *PHQ-9* Patient Health Questionnaire; *CES-D* Center for Epidemiologic Studies Depression scale; *WDEQ-A* Wijma Delivery Expectancy/Experience Questionnaire; *STAI* State-Trait Anxiety Inventory; *PSS-4* Perceived Stress Scale-4; *STR* Subjective Stress Scale (Schär et.al 1973) OSLO-3 = Oslo Social Support Scale (OSSS-3); *SSQ-6* Social Support Questionnaire - Short Form (SSQ6); *MSSI* Maternal Social Support Index; *DASH* Domestic Abuse, Stalking and Harassment; *AAS* Abuse assessment screens; *WAST* Woman Abuse Screening Tool; *NSDUH* National Survey on Drug Use and Health; *CAGE* Cut-Annoyed-Guilty-Eye; *HSI* The Heaviness of Smoking Index

In addition, 21 indicators appeared to be common and were grouped as follows: 1)Socio-demographic/Economic Status (SES: communication, age, place of birth, education, marital status, occupation, financial and housing situation), 2)medical factors (medical/obstetrics, medication use, unwanted/unplanned pregnancy, late follow-up), 3)mental state (depression, anxiety, psychological history, stress), 4)social situation (social support and domestic violence) and 5)substance use (drugs, alcohol and tobacco).

Note that the references mentioned in Table 1 can be found in additional file 1.

### Selection of the indicators, constructing the Born in Brussels ST – phase 2 of step 2

Table 1 illustrates the recurrent and relevant indicators. All indicators, except for ‘communication’, ‘age’, ‘place of birth’, ‘medical/obstetrics’ and ‘late follow-up’, were selected as recurrent indicators. The selected indicators ‘alcohol’, ‘drugs’ and ‘tobacco’ were grouped under one indicator ‘substance use’. This resulted in a selection of 14 indicators of the 21 indicators (Table 1). The further selection based on relevancy included ‘communication’ and excluded the following recurrent indicators: ‘unwanted/unplanned pregnancy’, ‘psychological history’ and ‘stress. This resulted in the Born in Brussels ST including 12 indicators which can be divided into 4 categories: SES (communication, place of birth, education, marital status, occupation, financial and housing situation), mental state (depression and anxiety), social situation (social support and domestic violence) and substance use (alcohol, drugs and tobacco).

### Literature search of questionnaires – phase 3 of step 1

Further literature search of indicator-specific questionnaires resulted in more than 23 questionnaires for mental state (anxiety and depressions), 17 questionnaires for social support (included in Table 2), 16 for domestic violence, 15 for substance use, and two antenatal guidelines (included in Table 2) that review psychosocial indicators. (Other results available upon request).

**Table 2:** Selection process of the items (i.e. questions) measuring the 13 indicators of antenatal psychosocial vulnerability (phase 4 in Fig. 1)

Indicators	Validated items based on guidelines (fig1: phase 4a)	Validated items based on scientific value or usability (Fig. 1: phase 4b)				Self-designed items (Fig. 1: phase 4c)	Number of items
–	Antenatal (inter) national guidelines	Validated questionnaires		Number of items ≤5	Used/Questioned in pregnant population	Item self-designed and/or based on	
Category: Socio-economic/demographics status							
Communication	–	–		–	–	Expert panels	**1**
Birth country	–	–		–	–	CEPIP (1)	**1**
Residence status^a^	–	–		–	–	Expert panels	**1**
Education	–	–		–	–	CEPIP (1)	**1**
Occupation	–	–		–	–	CEPIP (1)	**1**
Partner’s occupation^b^	–	–		–	–	CEPIP (1) & C.D.V.P (2)	**1**
Income	–	–		–	–	C.D.V.P (2)& Van Damme et.al (3)	**1**
Housing situation	–	–		–	–	Van Damme et.al (3) & Kind en Gezin kansarmoede criteria	**1**
Category: Mental state							
Depression	NICE Antenatal and postnatal mental health guideline (4)	Whooley (5)		2 items	Yes	–	**2**
Anxiety	NICE Antenatal and postnatal mental health guideline (4)	Generalised Anxiety Disorder (GAD-2) (6)		2 items	Yes	–	**2**
Category: Social situation							
Social support	–	• Maternity Social Support Scale • Oslo Social support scale (OSLO-3) • Norbeck Social Support Questionnaire • Social Support Questionnaire 6 (SSQ-6) • The Duke-UNC Functional Social Support Questionnaire (FSSQ) - 8 items	• Interpersonal Support Evaluation List • Social Support Questionnaire 27 items • Berlin Social Support Scales (BSSS) • Multidimensional Scale of Perceived Social Support (MSPSS)	• OSLO-3 • SSQ- 6	Oslo Social support scale (OSLO-3) (7)	–	**3**
Violence	–	• AAS Abuse Assessment Screen • CTS-2 Revised Conflict Tactics Scale • PVS Partner Violence Screen • OAS Ongoing Abuse Screen • HITS Hurt, Insulted, Threaten and Scream • STaT (slapped, threatened throw) • WAST Woman Abuse Screen Tool • HARK Humiliation, Afraid, Rape, Kick Screen	• ISA Index of Spouse Abuse • SVAWS Severity of Violence Against Women Scales • CAS Composite Abuse Scale • CAS Composite Abuse Scale short form • ABI Abusive Behaviour Inventory • PAS Partner Abuse Scale • MMEA Multidimensional Measure of Emotional Abuse	• AAS Abuse Assessment Screen • OAS Ongoing Abuse Screen • HITS Hurt, Insulted, Threaten and Scream	(3 of 5 items) OAS Ongoing Abuse Screen (8)	–	**3**
Category: Substance use							
Substance use (alcohol, smoking, drugs, and cannabis^a^)	Australian Guideline for Supporting Pregnant Women who use Alcohol or other Drugs: A Guide for Primary Health Care Professionals (9)	• IRIS Indigenous Risk Impact Screen • ASSIST V3 Alcohol, Smoking and Substance Use Involvement Screening Test Version 3 • FTND Fagerstrom Test for Nicotine Dependence • TLFB Timeline Follow Back		–	1 item of ASSIST v3 (10)	Expert panels	**4**
Total indicators: 13							**Total items: 22**
Informative Indicators							
Psychological history^a^	–	–		–	–	Expert panels	**1**
Medication use^a^	–	–		–	–	Expert panels	**1**

^a^ Indicator item added after refinement

^b^ Indicator marital status was adapted to partner’s occupation

Mentioned references can be found in additional file 2

### Selection of items, constructing the born in Brussels ST – phase 4 of step 2

Table 2 provides an overview of the quality appraisal and usability of the items for each of the 12 selected indicators. This evaluation resulted in the selection of 20 close-ended items, constituting thus the Born in Brussels ST, with either categories, dichotomous or rating scales as answer options.

Validated questionnaires were selected to measure the following indicators: social support (OSLO-3), mental state (Whooley for depression and Generalized Anxiety Disorder (GAD-2) for anxiety), domestic violence (Ongoing Abuse Screen (OAS)) and substance use (ASSIST v3). The 2-items Whooley questionnaire, for depression symptoms, questions the past month’s mood and interest. A positive screening requires at least one “yes” from the dichotomous answer options (Yes-No). The 2 items GAD-2 questionnaire identifies anxiety disorder symptoms experienced in the previous 2 weeks. A score of 3 or more on this 4-point Likert scale results in a positive screening. Furthermore, the 5-items OAS questionnaire measures different levels of ongoing intimate partner violence (i.e. physical, emotional, fear and sexual violence) with a dichotomous metric (yes-no). An affirmative screening requires at least one yes. The 3-itemsOSLO-3 questionnaire measures perceived support and social network with a rating scale. The ASSIST v3 questionnaire, with dichotomous and rating scales as answer options, was selected for its comprehensive substance use screening (i.e. alcohol, smoking and drugs use).

The SES items, with categorical answer options, are self-designed and derive from the national ‘Centre d’ Epidémiologie Périnatale’ (CEPIP) report, prenatal assessment forms used in regional maternal care (i.e. ONE and Kind&Gezin) and questionnaires from previous studies.

Note that we received agreement from all authors to use their questionnaire except from the Oslo3 from which we were unable to get in contact with the author. In addition, the references mentioned in Table 2 can be found in additional file 2.

### Born in Brussels ST: assigned points, thresholds and ST refinement – phase 5 and 6 of step 3

From the 482 surveys sent, 168 caregivers assigned weights to each indicator which resulted in means varying between 4.80 (SD = 2.41) and 9.01 (SD = 1.64) (Table 3). From the quartile distribution of this data, a score of 1, 2, 3 or 4 was attributed to an indicator. The indicators ‘place of birth’, ‘occupation of the partner’ and ‘occupation of the pregnant woman’ were attributed a score of 1. A score of 2 was attributed to ‘education’, ‘anxiety’ and ‘communication’. ‘Financial situation’, ‘depression’ and ‘social support’ received a score of 3. The highest score, 4, was attributed to ‘housing’, ‘substance use’ and ‘domestic violence’. The indicator ‘residence status’ was added later and received a score of 3, as proposed by the expert panels. Points were further attributed to each answer option. The total score on the tool was 30 and 33 after adding the indicator ‘residence status’. The indicators’ scores, assigned points and threshold values can be found in the additional file 3.

**Table 3:** Scores attributed to the indicators based on the survey’s result

		Weight attributed from survey	Score attribution	
	Indicators ordered from highest to lowest mean score	Mean (sd) scores (*N* = 168)	Attributed scores ^a^	Adapted Scores ^b^
1	Domestic violence	9.01 (sd = 1.64)	4	4
2	Substance use	8.46 (sd = 1.78)	4	4
3	Housing situation	8.34 (sd = 1.87)	4	4
4	Social support	8.02 (sd = 1.83)	3	3
5	Depression	8.12 (sd = 1.89)	3	3
6	Financial situation	7.93 (sd = 2.04)	3	3
7	Anxiety	7.14 (sd = 2.01)	2	2
8	Communication	6.79 (sd = 2.04)	2	2
9	Education	5.93 (sd = 2.92)	2	1
10	Occupation	5.92 (sd = 2.10)	1	1
11	Partner’s occupation	5.70 (sd = 2.03)	1	1
12	Place of birth	4.80 (sd = 2.41)	1	0.5
	Total score		30	28.5
13	Residence status ^c^	/	3	3
	Total score		33	31.5

^a^ Based on Quartile distribution: Q1 = 5,92; Q2 = 7,54; Q3 = 8,29

→Score 1 = mean scores ≤ Q1

→Score 2 = Q1 < mean scores ≤ Q2

→Score 3 = Q2 < mean scores ≤ Q3

→Score 4 = mean scores > Q3

^b^ Adjusted after experts ‘consult

^c^ Added after experts’ consult

In the final step, some content and score adaptations occurred based on the participants’ comments and expert panel’s revision. The indicator ‘residence status’ was added of which its item was designed by the expert panel. Indicators measuring ‘psychological history’, ‘medication use’ and ‘violence history’ were added as informative unscored indicators. These indicators were added to be alert for possible recurrences of past situations or (un)intentional misuse in the case of medication use. To be more inclusive, the indicator ‘marital status’ was replaced by ‘partner’s occupation’ from which the information about a partner’s presence or absence can be obtained. To cover other vulnerability factors than the ones in the tool, a comments section was also added.

Some further adaptations to the items followed. The selected item of the ASSIST v3 questionnaire for the substance use screening (i.e. alcohol, smoking and drugs use) was adapted to the antenatal period. In addition, cannabis was separated from ‘drugs’ as a single item on request of the expert panel who denoted the difference in care approach. In addition, the dichotomous metric of the item ‘violence’ has been replaced by the Likert scale of the HITS questionnaire to allow free confession of any level of violence [26].

Regarding the scores’ adaptations on the tool, the scores attributed to the indicators ‘birth country’ and ‘education’ were lowered from 1 to 0.5 and 2 to 1 respectively, since experts denoted their minor impact. Scores of ‘psychological history’, ‘medication use’ and ‘violence history’ were not included as the tool focuses on the recent situation (i.e. past two weeks or months). Moreover, determining medication misuse requires deeper investigation and medication knowledge, as explained by the experts and is therefore complex to attribute a score.

**Figure 2:**
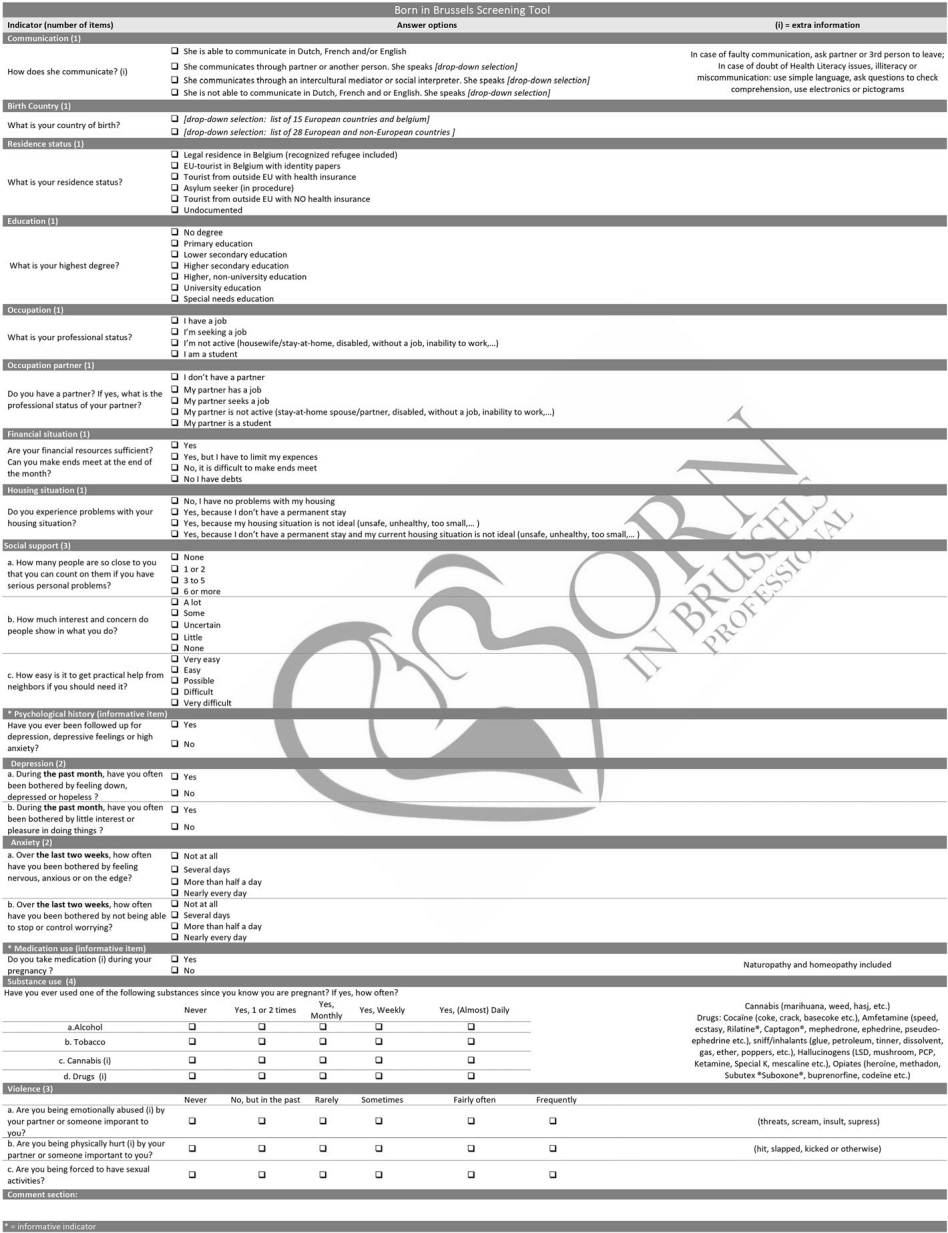
**Fig. 2** The Born in Brussels Screening Tool measures antenatal psychosocial vulnerability. The Born in Brussels Screening Tool focuses on 13 indicators (communication, place of birth, residence status, education, occupational status, partner’s occupation, financial situation, housing situation, social support, depression, anxiety, substance use and domestic violence) and hasa total of 22 items. In addition, two informative indicators (psychological history and medication use) are included in the Born in Brussels Screening Tool. A comment section is available for clarifying or reporting (other) vulnerability indicators

The adjustments made resulted in the Born in Brussels ST of 13 indicators, excluding the informative indicators, measurable by 22 items and resulting in a total score of 31.5 (Fig. 2).

## Discussion

Timely detection of vulnerable pregnant women is one of the main objectives of the healthcare system. In Belgium, the NIHDI has therefore initiated the Born in Brussels project. One of the objectives was to develop a screening tool, focused on antenatal psychosocial vulnerability. Based on literature review and insights from experts in the field, the content of the screening tool and the corresponding scores were determined. The tool focuses on 13 indicators: communication, place of birth, residence status, education, occupational status, partner’s occupation, financial situation, housing situation, social support, depression, anxiety, substance use and domestic violence and is measured by 22 items.

The indicators and items included in the Born in Brussels ST are those most often reported in literature and supported by experts in the field. The focus on both psychological and social indicators, explaining a form of antenatal vulnerability, is an approach also used by Vos et.al and Fline-barthes et al. [16, 19] amongst others (described in Table 2). However, more indicators can identify antenatal psychosocial vulnerability. Indicators such as the quality of the partner relation, teenage pregnancy and prenatal maternal stress (PMS) were not included, for example. It could be argued that they are covered through indicators that reflect the psychosocial situation in depth such as ‘social support’, ‘depression’, ‘anxiety’, ‘housing’, ‘income’ or ‘violence’. Moreover, based on Beydoun et.al description of the multidimensionality of PMS, PMS can be considered as being included in the Born in Brussels ST. He describes the multidimensionality of PMS as the result of “an imbalance between environmental demands (acute and chronic stressors) and individual resources (socio-economic conditions, life style, personality and social support), leading to a heightened stress perception and increased risk of maladaptive emotional responses (e.g. anxiety and depression)” [27].

In addition, initially excluded indicators were added upon refinement of the tool due to the particular Brussels Metropolitan context, research evidences and for research purposes. Studies provide evidence that immigrant mothers (i.e. women of foreign origin) have less access to prenatal care [28], due to language barriers [29] or residence status [30], and are at risks for worse pregnancy outcomes (e.g. low birth weight [31], perinatal mortality [32] or maternal morbidities [33]). Therefore, the indicators ‘communication (more specifically ‘language proficiency’)’, ‘residence status’ and ‘place of birth’, were included in the tool. The inclusion of these elements underpins the application of the Born in Brussels tool in other regions, especially Metropolitan areas, which aim to offer integrated care focused on psychosocial well-being.

The selected items for the Born in Brussels ST have either been applied or validated in a pregnant population group and most of them appear to be recommended by (antenatal) guidelines. The NICE guideline [34] recommends the Whooley as a pre-screening for depression during pregnancy and the EPDS for further assessment if there is a positive score on the Whooley questionnaire. The OSLO-3, which was the best option to measure social support for our tool, has been applied but not yet validated in a pregnant population. However, it allows for comparisons as it has been used in different European countries [35].

Although we believe that the Born in Brussels ST is relevant for use in practice, since this brief questionnaire encompasses the essential antenatal psychosocial vulnerability indicators, a few limitations need to be acknowledged. The tool does not include medical factors, which could have complemented the multidimensionality of a vulnerability-screening tool. Also, while many psychosocial indicators could be included, a selection of 13 indicators was made. Some relevant ones might have been overlooked; however, a longer instrument hampers the applicability in practice and reduces, as a result, the response rate [2, 36]. In addition, although solved with a comment section, the close-ended items do not cover all answer possibilities. Still, they facilitate the completion of a questionnaire and offer uniformity in different possible settings [37, 38]. Another limitation could be the replacement of dichotomous scales with more informative scales, which from a daily practice’s perspective provides more insights. Still, converting it back to the original scale remains possible and thus allows comparative analyses with other studies.

Regarding the attribution of the scores, in contrast to Vos et.al [19], that determined their indicators’ weights by odds ratios/relative risks of each indicator [19] the Born in Brussels ST indicators’ weights were determined by experts’ subjective rating. However, the overall result was similar, confirming the expertise of the surveyed experts and the decisions made.

A future and in depth validation analysis is still needed. Next to the evaluation of the subscales, it is relevant to explore the possibility to determine a total cut off score for antenatal psychosocial vulnerability during a validation phase.

In parallel, this study has important strengths. The methodology used for the construction of our questionnaire is in line with previous developments of screening tools (R4U, Mind2Care, PRO, ALPHA, AQ Lille-Roubaix) and similar to the approach suggested by Peterson et.al [37] and Jhangiani et.al [38] that guided us on what to consider when constructing our questionnaire. Moreover, the screening tool builds on the literature, experts’ advice and includes a selection of common and reliable antenatal psychosocial vulnerable indicators and items. The tool also largely complies with questionnaires’ design recommendations such as BRUSO (Brief, Relevant, Unambiguous, Specific and Objective). Thus, the Born in Brussels ST is one of the few thorough screening tools that is brief and item-sensitively-ordered to detect antenatal psychosocial vulnerability.

Developing a comprehensive screening tool measuring antenatal psychosocial vulnerability was the objective of this paper. However, it is acknowledged from the literature and care practices that other aspects need to be considered ensuing the development of a screening tool. One is that screening cannot be done without an associated care offer [39]. Therefore, it is believed that an associated integrated and personalised care path, gathering Brussels’ antenatal care and (social) care organisations is the next step needed. Moreover, an accompanying training for caregivers on how to screen for sensitive topics and avoid stigmatisation issues is also an aspect to promote [2, 22, 39]. Lastly, other studies [10, 40] highlight the importance to investigate the implementation process, the effectiveness of a screening tool, its psychometrics (i.e. validity, reliability and a defined cut-off value) and any associations with pregnancy and birth outcomes. Therefore, further research will be performed when implementing the Born in Brussels ST.

## Conclusion

The development of the Born in Brussels ST results from an elaborative literature research and experts’ involvement. From this, 22 brief and practical oriented items were developed that measure 13 indicators of antenatal psychosocial vulnerability. Introducing this tool in the caregiver’s current practices might increase the timely detection of vulnerable pregnancies, facilitate referrals, enable the set-up of appropriate prevention strategies and decrease the risk of adverse perinatal outcomes.

## Supplementary Information

**Additional file 1.** References of Table 1. Additional file 1 illustrates the references mentioned in Table 1.

**Additional file 2.** References of Table 2. Additional file 2 illustrates the references mentioned in Table 2.

**Additional file 3.**Scores of the Born in Brussels Screening Tool. Additional file 3 illustrates the scores of the Born in Brussels Screening Tool.

## Data Availability

The datasets used and/or analysed during the current study are available from the corresponding author on reasonable request.

## Abbreviations

ST: Screening Tool
EPDS: Edinburgh Postnatal Depression Scale
NIHDI: National Institute for Health and Disability Insurance
SSQ-6: Social Support Questionnaire - Short Form
SES: Socio-demographic/Economic Status
GAD: Generalized Anxiety Disorder
OAS: Ongoing Abuse Screen
CEPIP: Centre d’ Epidémiologie Périnatale
PMS: Prenatal Maternal Stress
BRUSO: Brief, Relevant, Unambiguous, Specific and Objective
NICE: National Institute for Health and Care Excellence
