# Supplementary material for: Born in Brussels screening tool: the development of a screening tool measuring antenatal psychosocial vulnerability
Source: BMC Public Health. 2021 Aug 6;21:1522. doi: 10.1186/s12889-021-11463-8 (PMC8348826; doi:10.1186/s12889-021-11463-8)
Supplement: Supplementary file 1 — Additional file 1. References of Table 1. Additional file 1 illustrates the references mentioned in Table 1. [file 12889_2021_11463_MOESM1_ESM.docx]

Supplementary file 2: References of table 1

1. Beeckman, K., Laubach, M., Spinnoy, A., De Koster, K., Almaci, Z., Linster, A. et al.. Kwetsbare zwangere, een perinataal zorgaanbod op maat. Brussel, België. 2016..

2. Zucca S, Lambotte I, Goban V, Fourneret P, Valderrama A, Iglesisas MH, et al. C.D.V.P. Carnet de Dépistage de la Vulnérabilité Périnatale.

3. Van Damme R, Van Parys AS, Vogels C, Roelens K, Lemmens GMD. A mental health care protocol for the screening, detection and treatment of perinatal anxiety and depressive disorders in Flanders. Journal of Psychosomatic Research: Elsevier Inc.; 2020.

4. EMBRACE: zorgpad voor kwetsbare zwangeren na detectie bezorgdheid in grootstad Antwerpen. Antwerpen; 2018.

5. Labbé É, Moulin JJ, Guéguen R, Sass C, Chatain C, Gerbaud L. Un indicateur de mesure de la précarité et de la « santé sociale » : le score EPICES. L'expérience des Centres d'examens de santé de l'Assurance maladie. La Revue de l'Ires. 2007;53(1):3-49.

6. Fline-Barthes MH, Vandendriessche D, Gaugue J, Urso L, Therby D, Subtil D. Dépistage des situations de vulnérabilité psychosociale et toxicologique pendant la grossesse: Évaluation d'un auto-questionnaire par comparaison aux données du dossier médical. Journal de Gynecologie Obstetrique et Biologie de la Reproduction. 2015;44(5):433-42.

7. Chanal C, Raffier L. Repérage des vulnérabilités en maternités à l’échelle d’une région : méthode, résultats et perspectives.

8. Chaplot Sp. Présentation d’un auto-questionnaire de repérage des vulnérabilités et des consommations de substances psycho-actives. Pays de Loire; 2015.

9. Quispel C, Schneider TAJ, Bonsel GJ, Lambregtse-Van Den Berg MP. An innovative screen-and-advice model for psychopathology and psychosocial problems among urban pregnant women: An exploratory study. Journal of Psychosomatic Obstetrics and Gynecology. 2012;33(1):7-14.

10. Vos AA, van Veen MJ, Birnie E, Denktaş S, Steegers EAP, Bonsel GJ. An instrument for broadened risk assessment in antenatal health care including non-medical issues. International Journal of Integrated Care. 2015;15(JAN-MAR 2015).

11. Ruf-Leuschner M, Pryss R, Liebrecht M, Schobel J, Spyridou A, Reichert M, et al. Preventing further trauma: KINDEX mum screen-assessing and reacting towards psychosocial risk factors in pregnant women with the help of smartphone technologies. 2013.

12. Curry MA, Campbell RA, Christian M. Validity and reliability testing of the prenatal psychosocial profile. Research in Nursing & Health. 1994;17(2):127-35.

13. Harrison PA, Sidebottom AC. Systematic prenatal screening for psychosocial risks. Journal of Health Care for the Poor and Underserved. 2008;19(1):258-76.

14. Goldenberg RL. Abbreviated scale for the assessment of psychosocial status in pregnancy: Development and evaluation. Acta Obstetricia et Gynecologica Scandinavica, Supplement. 1997;76(165):19-29.

15. Kohlhoff J, Hickinbotham R, Knox C, Roach V, Am BB. Antenatal psychosocial assessment and depression screening in a private hospital. 2015.

16. Austin MP, Colton J, Priest S, Reilly N, Hadzi-Pavlovic D. The antenatal risk questionnaire (ANRQ): Acceptability and use for psychosocial risk assessment in the maternity setting. Women and Birth. 2013;26(1):17-25.

17. Reid AJ, Biringer A, Carroll J, Midmer D, Wilson L, Chalmers B, et al. Using the ALPHA form in practice to assess antenatal psychosocial health. Antenatal Psychosocial Health Assessment. CMAJ : Canadian Medical Association journal = journal de l'Association medicale canadienne. 1998;159:677-84.

18. Matthey S, Phillips J, White T, Glossop P, Hopper U, Panasetis P, et al. Routine psychosocial assessment of women in the antenatal period: Frequency of risk factors and implications for clinical services. Archives of Women's Mental Health. 2004;7(4):223-9.

19. Austin MP, Hadzi-Pavlovic D, Saint K, Parker G. Antenatal screening for the prediction of postnatal depression: validation of a psychosocial Pregnancy Risk Questionnaire. Acta Psychiatrica Scandinavica. 2005;112(4):310-7.

20. Howard L, Hunt K, Slade M, O'Keane V, Senevirante T, Leese M, et al. Assessing the needs of pregnant women and mothers with severe mental illness: The psychometric properties of the Camberwell Assessment of Need - Mothers (CAN-M). International Journal of Methods in Psychiatric Research. 2007;16(4):177-85.
