# Supplementary material for: Born in Brussels screening tool: the development of a screening tool measuring antenatal psychosocial vulnerability
Source: BMC Public Health. 2021 Aug 6;21:1522. doi: 10.1186/s12889-021-11463-8 (PMC8348826; doi:10.1186/s12889-021-11463-8)
Supplement: Supplementary file 2 — Additional file 2. References of Table 2. Additional file 2 illustrates the references mentioned in Table 2. [file 12889_2021_11463_MOESM2_ESM.docx]

Supplementary file 3: References of table 2

1. Virginie Van L, Caroline D, Christian D, Charlotte L. Perinatale gezondheid in het Brussels Gewest Périnatalite PROGRAMME. 2017.

2. Zucca S, Lambotte I, Goban V, Fourneret P, Valderrama A, Iglesisas MH, et al. C.D.V.P. Carnet de Dépistage de la Vulnérabilité Périnatale.

3. Van Damme R, Van Parys AS, Vogels C, Roelens K, Lemmens GMD. A mental health care protocol for the screening, detection and treatment of perinatal anxiety and depressive disorders in Flanders. Journal of Psychosomatic Research: Elsevier Inc.; 2020.

4. Nccmh. NICE guideline. Antenatal and postnatal mental health: clinical management and service guidance. 2014.

5. Whooley MA, Avins AL, Miranda J, Browner WS. Case-finding instruments for depression: Two questions are as good as many. Journal of General Internal Medicine. 1997;12(7):439-45.

6. Kroenke K, Spitzer RL, Williams JBW, Monahan PO, Löwe B. Anxiety disorders in primary care: Prevalence, impairment, comorbidity, and detection. Annals of Internal Medicine. 2007;146(5):317-25.

7. Dalgard OS, Bjork S, Tambs K. Social support, negative life events and mental health. British Journal of Psychiatry. 1995;166(JAN.):29-34.

8. Ernst AA, Weiss SJ, Cham E, Marquez M. Comparison of three instruments for assessing ongoing intimate partner violence. Medical science monitor : international medical journal of experimental and clinical research. 2002;8(3):CR197-201.

9. Breen C, Awbery E, Burns L. Supporting pregnant women who use alcohol or other drugs: a review of the evidence | NDARC - National Drug and Alcohol Research Centre. 2014.

10. Humeniuk R, Ali R. Validation of the Alcohol, Smoking and Substance Involvement Screening Test (ASSIST) and Pilot Brief Intervention: A Technical Report of Phase II Findings of the WHO ASSIST Project. 2006. Report No.: 924159439X.
