# Supplementary material for: Born in Brussels screening tool: the development of a screening tool measuring antenatal psychosocial vulnerability
Source: BMC Public Health. 2021 Aug 6;21:1522. doi: 10.1186/s12889-021-11463-8 (PMC8348826; doi:10.1186/s12889-021-11463-8)
Supplement: Supplementary file 3 — Additional file 3.Scores of the Born in Brussels Screening Tool. Additional file 3 illustrates the scores of the Born in Brussels Screening Tool. [file 12889_2021_11463_MOESM3_ESM.pdf]

Screening tool Born in Brussels							
**Questions**	**Answer options**	(i) = extra information	**Step 1: determining the assigned points of the included validated questionnaires**	**Step 2: determining the cumulative score's cut-off value of the included validated questionnaire**	**Step 3a: Assigning points based on the indicator' score, determined from expert survey results**	**Step 3b: distributing the points over the answer options based on the**	**Step 4: determing threshold values deploying a care path and based on expert panels' input**
**Question 1: Communication**					**INDICATOR SCORE = 2**		
1.How does she communicate? (i)	❑ She is able to communicate in Dutch, French and/or English	In case of faulty communication, ask partner or 3rd person to leave; In case of doubt of Health Literacy issues, illiteracy or miscommunication: use simple language, ask questions to check comprehension, use electronics or pictograms'	N.A.	N.A.	0	Expert panels' input	≥1
	❑ She communicates through partner or another person. She speaks [drop-down menu]				1		
	❑ She communicates through an intercultural mediator or social interpreter.She speaks [drop-down menu]				1		
	❑ She is not able to communicate in Dutch, French and or English. She speaks [drop-down menu]				2		
**Question 2: Birth Country**		**Self-designed item from expert panels**			**INDICATOR SCORE = 0.5**		
2. What is your country of birth?	❑ [drop-down menu: list of EU 15 country and belgium]		N.A.	N.A.	0	CEPIP report's catogerisation	No care path
	❑ [drop-down menu: EU 28 and Non-EU country]				0,5		
**Question 3: Residence status**		**Self-designed item from expert panels**			**INDICATOR SCORE = 3**		
3. What is your residence status?	❑ Legal residence in Belgium (recognized refugee included)		N.A.	N.A.	0	Expert panels' input	≥0.5
	❑ EU-tourist in Belgium with identity papers				0		
	❑ Tourist from outside EU with health insurance				0		
	❑ Asylum seeker (in procedure)				0,5		
	❑ Tourist from outside EU with NO health insurance				3		
	❑ Undocumented				3		
**Question 4: Education**		**item from CEPIP report ***			**INDICATOR SCORE = 1**		
4. What is your highest degree?	❑ No degree		N.A.	N.A.	1	Research team	No care path
	❑ Primary education				1		
	❑ Lower secondary education				0,5		
	❑ Higher secondary education				0,5		
	❑ Higher , non-university education				0		
	❑ University education				0		
	❑ Special needs education				0,5		
**Question 5: Occupation**		**item from CEPIP report ***			**INDICATOR SCORE = 1**		
5. What is your professional status?	❑ I have a job		N.A.	N.A.	0	Research team	No care path
	❑ I'm seeking a job				1		
	❑ I'm not active (Housewife, disabled, without a job, inability to work,…)				1		
	❑ I am a student				1		
**Question 6: Occupation partner**		**item from CEPIP report & C.D.V.P ***			**INDICATOR SCORE = 1**		
6. Do you have a partner? If yes, what is the professional status of your partner?	❑ I don't have a partner		N.A.	N.A.	1	Research team	No care path
	❑ My partner has a job				0		
	❑ My partner seeks a job				1		
	❑ My partner is not active (Housewife, disabled, without a job, inability to work,…)				1		
	❑ My partner is a student				1		
**Question 7: Subjective income**		**item from C.D.V.P & Van Damme et.al ***			**INDICATOR SCORE = 3**		
7. Are your financial resources sufficient? Can you make ends meet at the end of the month?	❑ Yes		N.A.	N.A.	0	Research team	≥1
	❑ Yes, but I have to limit my expences				1		
	❑ No, it is difficult to make ends meet				2		
	❑ No I have debts				3		
**Question 8: Subjective housing situation**		**Item from Van Damme et.al & Kind en Gezin***			**INDICATOR SCORE = 4**		
8. Do you experience problems with your housing situation?	❑ No, I have no problems with my housing		N.A.	N.A.	0	Research team	≥2
	❑ Yes, because I don't have a permanent stay				2		
	❑ Yes, because my housing situation is not ideal (unsafe, unhealthy, too small,…)				2		
	❑ Yes, because I don't have a permanent stay and my current housing situation is not ideal (unsafe, unhealthy, too small,…)				4		
**Question 9: Social support**		**Item from Oslo-3 Questionnaire ***			**INDICATOR SCORE = 3**		
9a. How many people are so close to you that you can count on them if you have serious personal problems?	❑ None		1			category score distribution of the validated questionnaire	3
	❑ 1 or 2		2				
	❑ 3 to 5		3				
	❑ 6 or more		4				
9b. How much interest and concern do people show in what you do?	❑ A lot		5				
	❑ Some		4				
	❑ Uncertain		3	3–8	3–8 = 3		
	❑ Little		2	9–11	9–11 = 1,5		
	❑ None		1	12–14	12–14 = 0		
9c. How easy is it to get practical help from neighbors if you should need it?	❑ Very easy		5				
	❑ Easy		4				
	❑ Possible		3				
	❑ Difficult		2				
	❑ Very difficult		1				
**Question 10: Antecedent depression or anxiety disorder**		**Self-designed item from expert panels**			**INFORMATIVE INDICATOR**		
10. Have you ever been followed up for depression, depressive feelings or high anxiety?	❑ Yes		N.A.	N.A.	0	N.A	No care path
	❑ No				0		
**Question 11: Indication of depression**		**item from Whooley Questionnaire ***			**INDICATOR SCORE = 3**		
11a. During **the past month**, have you often been bothered by feeling down, depressed or hopeless ?	❑ Yes		1			dichotomous distribution of the validated questionnaire	3
	❑ No		0	<1	<1 = 0		
11b. During **the past month**, have you often been bothered by little interest or pleasure in doing things ?	❑ Yes		1	≥1	≥1 = 3		
	❑ No		0				
**Question 12: Indication of Anxiety**		**Item from GAD-2 Questionnaire ***			**INDICATOR SCORE = 2**		
12a. Over **the last two weeks**, how often have you been bothered by feeling nervous, anxious or on the edge?	❑ Not at all		0			dichotomous distribution of the validated questionnaire	2
	❑ Several days		1				
	❑ More than half a day		2				
	❑ Nearly every day		3	<3	<3 = 0		
12b. Over **the last two weeks**, how often have you been bothered by not being able to stop or control worrying?	❑ Not at all		0	≥3	≥3 = 2		
	❑ Several days		1				
	❑ More than half a day		2				
	❑ Nearly every day		3				
**Question 13: Medication use**		**Self-designed item from expert panels**			**INFORMATIVE INDICATOR**		
13. Do you take medication (i) during your pregnancy ?	❑ Yes	Naturopathy and homeopathy included	N.A.	N.A.	0	N.A	Care path if yes selected
	❑ No				0		
**Question 14: Substance use**		**Item from Assist v3 Questionnaire ***			**INDICATOR SCORE = 4**		
14. Have you ever used one of the following substances since you know you are pregnant? If yes, how often?		Cannabis (marihuana, weed, hasj, etc.)		N.A.		Research team based on the frequency scale distribution	≥2
	Never / Yes, 1 or 2 times / Yes, Monthly / Yes, Weekly / Yes, (Almost) Daily	Drugs: Cocaïne (coke, crack, basecoke etc.), Amfetamine (speed, ecstasy, Rilatine®, Captagon®, mephedrone, ephedrine, pseudeo-ephedrine etc.), sniff/inhalants (glue, petroleum, tinner, dissolvent, gas, ether, poppers, etc.), Hallucinogenen (LSD, mushroom, PCP, Ketamine, Special K, mescaline etc.), Opiaten (heroïne, methadon, Subutex ®Suboxone®, buprenorfine, codeïne etc.)	Never = 0		0 = 0		
a.Alcohol	❑ ❑ ❑ ❑ ❑		Yes, 1 or 2 times = 1		1 = 2		
b. Tobacco	❑ ❑ ❑ ❑ ❑		Yes, monthly = 2		2 = 3		
c. Cannabis (i)	❑ ❑ ❑ ❑ ❑		Yes, weekly = 3		≥3 = 4		
d. Drugs (i)	❑ ❑ ❑ ❑ ❑		Yes (almost) daily = 4				
**Question 15: Violence**		**Item from OAS & HITS questionnaire ***			**INDICATOR SCORE = 4**		
	Never / Yes, but in the past / Rarely / Sometimes / Fairly often / Frequently		Never = 0	N.A.		Research team based on the frequency scale distribution	≥2
15a. Are you being emotionally abused (i) by your partner or someone imporant to you?	❑ ❑ ❑ ❑ ❑ ❑	(threats, scream, insult, supress)	No, but in the past = 0		0 = 0		
15b. Are you being physically hurt (i) by your partner or someone important to you?	❑ ❑ ❑ ❑ ❑ ❑	(hit, slapped, kicked or otherwise)	Rarely = 1		1 = 2		
			Sometimes = 2		2 = 3		
15c. Are you being forced to have sexual activities?	❑ ❑ ❑ ❑ ❑ ❑		Fairly often = 3		≥3 = 4		
			Frequently = 4				
		*** references can be found in manuscript table 2**			**TOTAL SCORE = 31,5**		

Remarks:

Contact information: borninbrussels@uzbrussel.be Katrien.Beeckman@uzbrussel.be Kelly.Amuli@uzbrussel.be Kim.Decabooter@uzbrussel.be Anne.Renders@uzbrussel.be Florence.Talrich@uzbrussel.be
